# Colorectal cancer risk after removal of polyps in fecal immunochemical test based screening

**DOI:** 10.1016/j.eclinm.2023.102066

**Published:** 2023-07-05

**Authors:** D.E.F.W.M. van Toledo, J.E.G. IJspeert, M.C.W. Spaander, I.D. Nagtegaal, M.E. van Leerdam, I. Lansdorp-Vogelaar, E. Dekker

**Affiliations:** a Department of Gastroenterology and Hepatology, Amsterdam University Medical Centers, University of Amsterdam, Amsterdam, the Netherlands; b Amsterdam Gastroenterology, Endocrinology & Metabolism, Amsterdam, the Netherlands; c Cancer Center Amsterdam, Amsterdam University Medical Centers, Location Academic Medical Center, Amsterdam, the Netherlands; d Department of Gastroenterology and Hepatology, Erasmus MC University Medical Center, Rotterdam, the Netherlands; e Department of Pathology, Radboud University Medical Center, Nijmegen, the Netherlands; f Department of Gastroenterology, Netherlands Cancer Institute-Antoni Van Leeuwenhoek, Amsterdam, the Netherlands; g Department of Gastroenterology and Hepatology, Leiden University Medical Center, Leiden, the Netherlands; h Department of Public Health, Erasmus MC University Medical Center, Rotterdam, the Netherlands

**Keywords:** Colorectal cancer, Polypectomy, Screening, Prevention

## Abstract

**Background:**

Colonoscopy surveillance intervals are based on the predicted risk of metachronous colorectal cancer (CRC) after polyp removal. However, risk estimation per polyp subtype is difficult due to the fact that many patients have multiple polyps. To enable risk estimation per polyp subtypes we examined the metachronous CRC risk of subgroups based on presence or absence of co-occurring findings.

**Methods:**

Using high-quality screening colonoscopies performed after a positive fecal immunochemical test between 2014 and 2020 within the Dutch CRC screening program, we applied Cox regression analysis to evaluate the association between findings at baseline colonoscopy and metachronous CRCs. For our primary outcome, we appointed each patient to unique subgroups based on removed polyp subtypes that were present or absent at baseline colonoscopy and used the groups without polyps as reference. High-risk subgroups were individuals with high-risk serrated polyps, defined as serrated polyp ≥10 mm, sessile serrated lesions with dysplasia, or traditional serrated adenomas, as well as high-risk adenomas, defined as adenoma ≥10 mm or containing high-grade dysplasia.

**Findings:**

In total 253,833 colonoscopies were included. Over a median follow-up of 36 months (IQR, 21–57), we identified 504 metachronous CRCs. Hazard ratios for metachronous CRC was 1.70 (95% CI, 1.07–2.69) for individuals with high-risk serrated polyps without high-risk adenomas, 1.22 (0.96–1.55) for individuals with high-risk adenomas without high-risk serrated polyps, and 2.00 (1.19–3.39) for individuals with high-risk serrated polyps and high-risk adenomas, compared to patients without polyps.

**Interpretation:**

Accounting for co-occurring findings, we observed an increased metachronous CRC risk for individuals that had high-risk serrated polyps with the presence of high-risk adenomas, or individuals with high-risk serrated polyps without high-risk adenomas. These findings could provide more evidence to support post-polypectomy surveillance guidelines.

**Funding:**

None.

**Research in context**

**Evidence before this study**

We searched Pubmed, Cochrane, and MEDLINE for prospective and retrospective studies published from January 1, 2004 to October 2022, using the terms “colorectal cancer risk”, “metachronous risk”, “polypectomy”, “polyps”.

After resection of high-risk polyps, individuals have an increased risk of colorectal cancer. As such, the timing of colonoscopy surveillance is based on the presence of high-risk polyps at baseline colonoscopy. Risk estimation per polyp is rather difficult though in cases that have multiple polyps at baseline colonoscopy.

**Added value of this study**

In this study we aimed to estimate the risk for metachronous colorectal cancer per polyp subtype while accounting for the presence and/or absence of other polyps in a quality-assured surveillance setting of the Dutch screening program.

Individuals with high-risk serrated polyps without co-occurring high-risk adenomas had an increased risk to develop colorectal cancer. Strikingly, individuals with only high-risk adenomas did not show an increased risk for metachronous colorectal cancer, while individuals with both high-risk serrated polyps as well as high-risk adenomas had the highest risk.

**Implications of all the available evidence**

Results of this study could contribute to establish more restrictive polyp surveillance guidelines in a quality-assured setting.

## Introduction

Colorectal cancer (CRC) is the third most commonly diagnosed cancer in the world and second most common cause of cancer-related death.^1^ Endoscopic resection of CRC precursors is an effective method to reduce CRC related death.^2^ Surveillance after polypectomy might further reduce the risk of CRC. However, guidelines state that supporting evidence for surveillance intervals are moderate at best, affirming that there is a scientific knowledge gap about the risk magnitude in individuals developing CRC after removal of adenomas or serrated polyps.^3^, ^4^, ^5^, ^6^

A major obstacle for studies to evaluate metachronous CRC risk is that very large randomized controlled trials and/or large databases as well as a relatively long follow-up time are necessary. For this reason, most studies are retrospective evaluations of outdated data with restricted endpoints due to the absence of essential parameters like interval between baseline colonoscopy and CRC, size and subtype of polyps.^7^, ^8^, ^9^, ^10^, ^11^, ^12^ These retrospective studies have major limitations. For instance, quality of colonoscopy has improved over time due to the awareness and implementation of quality parameters (e.g. cecal intubation rate, adequacy of bowel preparation and adenoma detection rate), as well as use of high-definition endoscopes and advanced endoscopic imaging. Outdated studies also lack often to incorporate the interval between baseline colonoscopy and detection of CRC, resulting in simplified statistical methods like logistic regression only. Furthermore, recent literature has demonstrated the contribution of serrated polyps in CRC development.^13^^,^^14^ As data on serrated polyps were usually not reliably collected in the past, most retrospective studies do not have appropriate data on serrated polyps available. This hampers not only the evaluation of the post-polypectomy risk of serrated polyps, but also the possibility to correct for co-existing serrated polyps when evaluating the risk of adenomas. These issues urge the need for large prospective cohort evaluations to be able to assess the metachronous CRC risk in individuals with serrated polyps and/or conventional adenomas, and to analyse subgroups stratified for the presence of co-existing findings at baseline screening colonoscopy.

Aiming to investigate the metachronous CRC risk after detection and resection of all relevant polyp subtypes, we evaluated the risk of patient groups with and without co-existing findings using data of high-quality colonoscopies performed for a positive fecal immunochemical test (FIT) result in the setting of the Dutch CRC screening program.

## Methods

### Study design

This is a population based cohort study including baseline colonoscopies from the Dutch national CRC screening program and all CRC cases from the National Cancer Registration. All included baseline colonoscopies were screening colonoscopies of individuals that had a positive FIT. The first part of the study period the cut-off for positivity was ≥15 and after 6 months this was increased to ≥47 μg fecal haemoglobin per gram feces [f-Hb μg/g].

### Ethics statement

This study protocol was approved by the population screening research committee of the governmental National Institute for Public Health and the Environment due to its non-interventional nature. The privacy of participants was warranted by allocating participants to pseudonyms before data transfer to our research team, according to the General Data Protection Regulation Act.^15^

### Inclusion/exclusion criteria

We included all colonoscopies performed within the study period stretching from the start of the screening program in January 2014 to December 2020. At baseline, colonoscopies were excluded in case of uncertainty regarding polyp detection or complete polyp removal: no cecal intubation, insufficient bowel preparation, incomplete examination, referral for CT colonography, follow-up polypectomy or treatment of cancer, polyp was sent for pathological evaluation but pathology report was missing at random. We also excluded colonoscopies in which CRC was detected during baseline colonoscopy or within 6 months after baseline colonoscopy, since these cancers were assumed to be already present at baseline colonoscopy. In addition, colonoscopies were excluded when having a subsequent follow-up of less than 6 months.

### Data collection

We retrieved colonoscopy and pathology data from the national screening information system (ScreenIT). This data was prospectively registered from the start of the screening program in 2014 and linked to data regarding CRC cases that were received from The Netherlands Comprehensive Cancer Organization (IKNL) using the Netherlands Cancer Registry. No data was available of death or emigration of individuals.

### Outcome definitions and statistical analyses

High-risk serrated polyps were defined as any serrated polyp ≥10 mm, sessile serrated lesion (SSL) with dysplasia or traditional serrated adenoma (TSA). High-risk adenomas were defined as adenoma ≥10 mm, adenoma with high-grade dysplasia (HGD) or the presence of ≥5 low-risk adenomas. These polyp features were chosen as being high-risk, since each of the characteristics warrants strict surveillance according to the most recent ESGE post-polypectomy surveillance guideline.^3^ For this reason, villous component was not taken into account as high-risk feature. As primary outcome we analysed the metachronous CRC risk after resection of predefined polyp subtypes, taking into account the presence of other polyps at baseline colonoscopy (model 1). Each patient could only be assigned to one of the groups. Following groups were defined.

1) hyperplastic polyps <10 mm (no coexisting SSL, TSA or adenoma),
2) low-risk SSL (no dysplasia, <10 mm) without adenomas,
3) 1–4 low-risk adenomas (no HGD, <10 mm) without coexisting SSLs),
4) low-risk SSLs with low-risk adenomas,
5) high-risk serrated polyps without high-risk adenomas,
6) high-risk adenomas without high-risk serrated polyps,
7) high-risk serrated polyps with high-risk adenomas.

Using this model, the metachronous CRC risk of each polyp or polyp combination could be assessed, not being biased by the synchronous presence of another finding. The metachronous CRC risk of the individuals in each group were compared with those individuals without any polyp at baseline colonoscopy and adjusted for age at baseline colonoscopy and sex. Information on potential other demographic predictors were not available in our database. Adjusting for endoscopic centre was not deemed necessary because we did not expect this variable to affect the risk of CRC within our study population.

As secondary outcome, we assessed the metachronous CRC risk of predictors based on unique polyp characteristics found at baseline colonoscopy (e.g. polyp location, number of polyps and the presence of an unique high-risk polyp feature) (model 2). Each patient could be assigned to multiple groups. Both univariate and multivariate analyses were performed, in the latter we adjusted for sex, age at index colonoscopy, and predictors found at baseline colonoscopy. Only those predictors were included in multivariate model that were significant at univariate analysis because of relative low case frequencies in some subgroups.

For both models a cox proportional hazard analysis was used, reporting the hazard ratio (HR) with 95% confidence interval (CI). Risk estimation were not deemed reliable and not performed if CRC cases counted 5 or less per subgroup. A HR >1.5 was considered clinically relevant. The time to CRC or end of follow up was calculated from the date of colonoscopy until the date of CRC diagnosis, or end of follow up at December 2020. Comparable analyses were performed, in which the risk of metachronous CRC was assessed for proximal and distal CRC separately. Proximal cancers included those located from cecum to splenic flexure. Distal cancers included those located from descending colon to rectum. For each analysis, lesion size was based on the histopathological specimen. Polyps located in the cecum, ascending colon, hepatic flexure, transverse colon or splenic flexure were classified as proximal. Polyps located in the descending colon, sigmoid, or rectosigmoid were classified as distal, and polyps located in the rectum or rectosigmoid junction were classified as rectal.

We performed a sensitivity analysis for model 1 and model 2 in which all CRCs diagnosed within 12 months after baseline colonoscopy were excluded from analysis. A sensitivity analysis concerning the change of FIT cut-off concentration during the study period was considered but not deemed of additional value since we observed in a previous study that a different FIT cut-off did not affect detection rates of high-risk serrated polyps or high-risk adenomas within the same study population.^16^

Categorical variables were presented as count with proportion and continuous variables as median with interquartile range (IQR) when following a non-normal distribution. A p-value of <0.05 was considered statistically significant. We used IBM SPSS Statistics version 28.0.0.1 to perform the analyses.

### Role of the funding source

No particular funding from any commercial or public organisation was received for this study. All authors had full access to the data and approved the manuscript for publication.

## Results

### Baseline characteristics

**Fig. 1:**
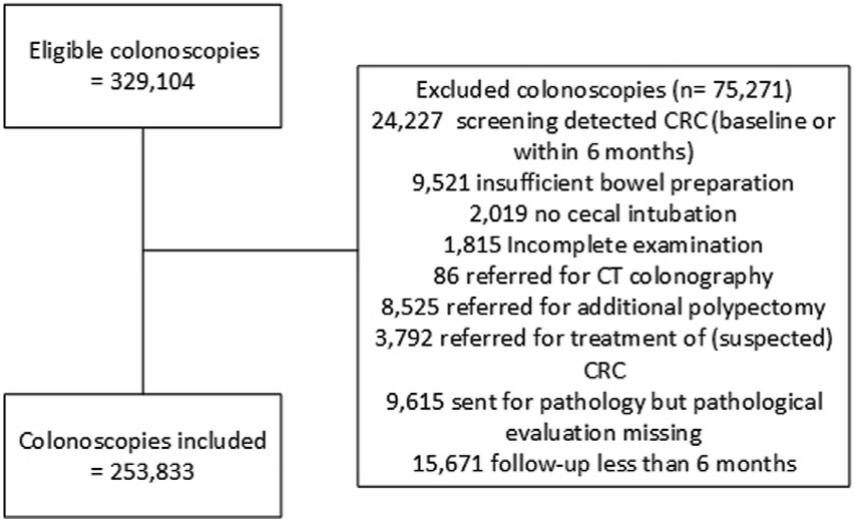
**Flowchart**.

The 253,833 included individuals (Fig. 1) had a median age of 69 years, and 105,511 (41.6%) were female. In total 78,068 (27.8%) individuals had no polyps at baseline colonoscopy, at least one serrated polyp was detected in 64,223 (25.3%) individuals, and at least one conventional adenoma was identified in 167,545 (66.0%) (Table 1). In total 9829 (3.9%) individuals had a large serrated polyp (≥10 mm), 2911 (1.1%) had a SSL with dysplasia, and 2125 (0.8%) had a TSA. Focusing on adenomas, 69,331 (27.3%) individuals had a large adenoma (≥10 mm), 9796 (3.9%) had an adenoma with high-grade dysplasia, and 5815 (2.3%) had 5 or more low-risk adenomas. All individuals together had in total 558,037 polyps; 360,438 (64.6%) tubular adenomas, 82,925 (14.9%) hyperplastic polyps, 74,834 (13.4%) tubulovillous adenomas, 33,468 (6.0%) SSLs, 3906 (0.7%) villous adenomas, and 2466 (0.4%) TSAs. More details are shown in Table 2.

**Table 1:** Characteristics of individuals, endoscopy centre and findings at baseline colonoscopy.

	Number (%)
**FIT-positive individuals undergoing colonoscopy**	253,833
Female	105,511 (41.6)
Age, median years (IQR)	69 (63–72)
**Endoscopy centre**	
Academic	7854 (3.1)
Non-academic	196,158 (77.3)
Private practice	49,821 (19.6)
Median duration of follow-up, months	36 (21–57)
**Findings at baseline colonoscopy**	
**No polyp**	70,468 (27.8)
**At least one serrated polyp and adenoma**	48,264 (19.0)
**At least one serrated polyp**	64,223 (25.3)
≥1 hyperplastic polyp	49,262 (19.4)
≥1 sessile serrated lesion	21,357 (8.4)
≥1 sessile serrated lesion with dysplasia	2911 (1.1)
≥1 traditional serrated adenoma	2125 (0.8)
Serrated polyp <10 mm	38,948 (15.3)
Serrated polyp ≥10 mm	9829 (3.9)
Serrated polyp of unknown size	28,137 (11.1)
1–4 low-risk sessile serrated lesion(s)	20,850 (8.2)
≥5 low-risk sessile serrated lesions	507 (0.2)
**At least one adenoma**	167,545 (66.0)
≥1 tubular adenoma	145,028 (57.1)
≥1 tubulovillous adenoma	54,806 (21.6)
≥1 villous adenoma	3427 (1.4)
≥1 adenoma with high grade dysplasia	9796 (3.9)
Adenoma <10 mm	100,076 (39.4)
Adenoma ≥10 mm	69,331 (27.3)
Adenoma of unknown size	77,928 (30.7)
1–4 low-risk adenoma(s)	93,715 (36.9)
≥5 low-risk adenomas	5815 (2.3)

**Table 2:** Polyp characteristics at baseline colonoscopy.

	Hyperplastic polyp	Sessile serrated lesion	Traditional serrated adenoma	Tubular adenoma	Tubulovillous adenoma	Villous adenoma
	82,925 (14.9)	33,468 (6.0)	2466 (0.4)	360,438 (64.6)	74,834 (13.4)	3906 (0.7)
**Size, n (%)**						
<10 mm	47,393 (8.5)	15,985 (2.9)	620 (0.1)	181,616 (32.5)	15,265 (2.7)	350 (0.1)
≥10 mm	3727 (0.7)	5962 (1.1)	1070 (0.2)	45,076 (8.1)	39,506 (7.1)	2289 (0.4)
Undefined	31,805 (5.7)	11,521 (2.1)	776 (0.1)	133,746 (24.0)	20,063 (3.6)	1267 (0.2)
**Location of polyps, n (%)**						
Proximal	20,610 (3.7)	21,498 (3.9)	511 (0.1)	164,520 (29.5)	15,723 (2.8)	676 (0.1)
Distal	32,679 (5.9)	7073 (1.3)	1052 (0.2)	130,949 (23.5)	35,460 (6.4)	1675 (0.3)
Rectum	18,551 (3.3)	1790 (0.3)	468 (0.1)	23,171 (4.2)	9600 (1.7)	574 (0.1)
Undefined	11,085 (2.0)	3107 (0.6)	435 (0.1)	41,798 (7.4)	14,051 (2.5)	981 (0.2)
**Histologic dysplasia, n (%)**						
LGD	0	3817 (0.7)	1964 (0.4)	355,099 (63.6)	68,429 (12.3)	3347 (0.6)
HGD	0		127 (0.0)	4332 (0.8)	5916 (1.1)	524 (0.1)

LGD low-grade dysplasia, HGD high-grade dysplasia.

### Follow-up data

During a median follow-up duration of 36 months (IQR, 21–57), 504 CRCs were identified; 267 (53.0%) in the proximal colon, 226 (44.8%) in the distal colon, and in 11 (2.2%) cases the location was undefined.

### Model 1: subgroups by presence/absence of other findings

In the subgroups, the incidence rate of CRC (cases per 1000 person years of follow up) was 0.53 for individuals without any polyp, 0.32 for individuals with only HPs<10 mm, 0.20 for individuals with only low-risk SSLs, 0.40 for individuals with only low-risk adenomas, 0.66 for individuals with low-risk SSLs and 1–4 low-risk adenomas, 0.68 for individuals with only high-risk adenomas, 0.89 for individuals with only high-risk serrated polyps, and 1.08 for individuals with both high-risk serrated polyps and high-risk adenomas (Table 3). Compared with individuals without any polyps, only those individuals with high-risk serrated polyps (HR 1.702, 1.078–2.686) or high-risk serrated polyps as well as high-risk adenomas (HR 2.003, 1.185–3.387) had an increased risk of metachronous CRC. Individuals with only low-risk adenomas, and individuals with only high-risk adenomas did not show an increased risk for metachronous CRC. Risk estimates were not calculated for individuals with only HPs<10 mm, low-risk SSLs, low-risk SSLs with low-risk adenomas because of the low frequency of CRC.

**Table 3:** Risk for metachronous CRC after detection and removal of polyp(s) at baseline colonoscopy analysed for co-occurrence of other polyp subtypes.

	Number of individuals	Number of CRC cases	Cases per 1000 person-years of follow-up	Unadjusted HR	p-value	Adjusted HR^a^	p-value
No polyps	70,468	115 (0.2%)	0.53	ref		ref	
HP <10 mm, without SSLs, TSAs, or adenomas	5299	5 (0.1%)	0.32	0.629 (0.257–1.541)	0.311	NA	NA
Low-risk SSL without adenomas	1865	1 (0.1%)	0.20	0.414 (0.058–2.966)	0.380	NA	NA
Low-risk adenomas without low-risk SSL	38,906	48 (0.1%)	0.40	0.773 (0.552–1.083)	0.135	0.771 (0.549–1.082)	0.132
Low-risk adenomas with low-risk SSL	1708	3 (0.2%)	0.66	1.381 (0.439–4.346)	0.581	NA	NA
High-risk adenomas without high-risk serrated polyps	70,647	171 (0.2%)	0.68	1.218 (0.962–1.544)	0.102	1.222 (0.961–1.554)	0.101
High-risk serrated polyps without high-risk adenomas	7829	22 (0.3%)	0.89	1.679 (1.064–2.650)	0.026	1.702 (1.078–2.686)	0.022
High-risk serrated polyps with high-risk adenomas	4204	16 (0.4%)	1.08	1.917 (1.136–3.236)	0.015	2.003 (1.185–3.387)	0.010

HP: Hyperplastic polyp, SSL: Sessile serrated lesion, TSA: Traditional serrated adenoma.

a Adjusted for age at baseline colonoscopy and sex.

### Model 2: multivariate analysis

Results of the multivariate analysis demonstrated an increased risk for individuals with any proximal adenoma (HR 1.360, 1.130–1.637), any villous adenoma (HR 2.069, 1.341–3.190), any TSA (HR 2.089, 1.144–3.816), any adenoma with HGD (HR 2.059, 1.529–2.771), or an older age at baseline colonoscopy (HR 1.058, 1.041–1.074) (Table 4). There was no increased risk for individuals with any proximal serrated polyp (HR 1.182, 0.906–1.542), tubulovillous adenoma (HR 1.079, 0.844–1.317), SSL with dysplasia (HR 1.307, 0.803–2.128), 5 or more low-risk adenomas (HR 1.459, 0.924–2.304), or females (HR 1.036, 0.865–1.241).

**Table 4:** Multivariate Risk for metachronous CRC after removal of polyp(s) at baseline colonoscopy adjusted for presence of polyps.

	Number of individuals	Number of CRC	Cases per 1000 person-years of follow-up	Univariate HR (95% CI)	p-value	Multivariate HR (95% CI)^a^	p-value
HP <10 mm	30,580	72	0.74	1.281 (0.998–1.644)	0.052		
SSL <10 mm, no dysplasia	10,521	19	0.63	1.098 (0.694–1.736)	0.690		
SSL low-risk 1–4	10,393	19	0.64	1.113 (0.704–1.760)	0.648		
SSL low-risk ≥5	128	0		NA	NA		
Low-risk adenoma 1–4	93,715	178	0.59	0.980 (0.817–1.177)	0.831		
Low-risk adenoma 3–4	15,439	34	0.53	1.191 (0.841–1.687)	0.325		
Proximal serrated polyp	29,161	70	0.78	1.357 (1.054–1.746)	0.018	1.182 (0.906–1.542)	0.226
Proximal adenoma	86,178	223	0.79	1.559 (1.307–1.858)	<0.001	1.360 (1.130–1.637)	0.001
Tubular adenoma	145,028	307	0.64	1.133 (0.947–1.355)	0.171		
Tubulovillous adenoma	54,806	152	0.76	1.286 (1.063–1.556)	0.010	1.079 (0.884–1.317)	0.456
Villous adenoma	3427	22	1.64	2.591 (1.690–3.973)	<0.001	2.069 (1.341–3.190)	0.001
**High-risk polyps**							
Serrated polyp ≥10 mm	9829	27	0.84	1.407 (0.955–2.073)	0.085		
SSL with dysplasia	2911	11	1.14	1.897 (1.043–3.447)	0.036	1.467 (0.789–2.726)	0.226
Traditional serrated adenoma	2125	11	1.48	2.424 (1.334–4.406)	0.004	2.089 (1.144–3.816)	0.017
Adenoma ≥10 mm	69,331	159	0.64	1.026 (0.850–1.238)	0.789		
Adenoma with HGD	9796	53	1.45	2.420 (1.821–3.218)	<0.001	2.059 (1.529–2.771))	<0.001
≥5 low-risk adenomas	5815	20	1.11	1.906 (1.219–2.981)	0.005	1.459 (0.924–2.304)	0.105

HP: Hyperplastic polyp, SSL: Sessile serrated lesion, HGD: High-grade dysplasia.

a Adjusted for age at baseline colonoscopy, sex, and other significant subgroups at univariate analysis.

An additional detailed univariate analysis of the subgroup with any adenomas ≥20 mm showed no increased risk (HR 1.040, 0.727–1.488). Univariate risk estimates were not calculated for individuals with ≥5 low-risk SSLs because no CRCs were observed in this subgroup.

### Sensitivity analyses

Separate evaluation of the risk for proximal CRCs showed comparable results in multivariate analysis and an increased risk for females (HR 1.399, 1.097–1785) or age (HR 1.068, 1.045–1.092) (Supplementary Table S1). Univariate analysis showed an increased metachronous risk for all high-risk serrated subgroups, but not for adenomas ≥10 mm or 5 or more low-risk adenomas. For distal CRC, multivariate analysis only showed an increased risk for individuals with any adenoma with HGD (HR 2.758, 1.743–4.362), or age (HR 1.048, 1.024–1.073), and a decreased risk for females (HR 0.688, 0.519–0.913) (Supplementary Table S2). Univariate showed only an increased risk for adenomas with HGD and 5 or more low-risk adenomas.

In another sensitivity analysis, additional exclusion of CRCs diagnosed within 6–12 months after baseline colonoscopy did not significantly affect the results of our primary and secondary analyses. Results are shown in Supplementary Table S3 and S4.

## Discussion

In this prospective cohort of 253,833 colonoscopies from FIT-positive individuals, we observed that those individuals with high-risk serrated polyps without co-existing high-risk adenomas, and those with both high-risk serrated polyps and high-risk adenomas were at increased risk to develop CRC at follow up. A multivariate analyses demonstrated that a clinically relevant increased metachronous CRC risk was mainly caused by having any TSA, villous adenoma, or adenoma with HGD at baseline colonoscopy. As such, size larger than 10 mm for both serrated polyps as adenomas was not confirmed as independent risk factor. Another interesting observation was that individuals with serrated polyps had a marked higher risk to develop proximal than a distal CRC.

This study is one of the largest studies on post-polypectomy CRCs in recent and prospectively collected colonoscopy and pathology data. Essential for these analyses, and scarce in other screening programs, was the consequent and structured registration of serrated polyps in our registry from the start of the screening program onwards. A major strength of our study and distinctive from most other studies is the fact that we appreciated the presence of co-existing relevant lesions as potential contributing factor to the metachronous risk of CRC (model 1). This method enabled us to address subsequent CRC risk with higher certainty to the polyp subtype of interest. Although this method resulted in some groups to limited CRC cases which would lead to unreliable risk estimations, a lower incidence per 1000 person-years of follow-up than the group without polyps is also deemed suggestive for a relatively low CRC risk. In contrast, the reported risk in the second model could also be attributed to co-occurring findings, although we adjusted for presence of significant subgroups. Because our results rely on high colonoscopy and pathology quality, we excluded low-quality colonoscopies and besides, it should be recognized that only accredited endoscopists who showed to have relative high detection rates for ADR and PSPDR,^17^ as well as accredited pathologists who performed an e-learning on serrated polyp diagnosis^18^ are allowed to perform in our screening program.

When interpreting our results, the relatively short follow up of three years (median of 36 months (IQR, 21–56) could be regarded as a limitation. The CRC incidence in each of the subgroups was slightly lower as compared to other studies, while the incidence in our reference group of individuals without any polyp was comparable to other studies.^7^^,^^8^ On the other hand, this short follow-up minimized the influence of subsequent surveillance colonoscopies (with resection of polyps) on the estimated HR of each of the polyp features. In fact, not accounting for surveillance endoscopies is a major limitation of 10-year follow-up studies that have been published previously.^7^^,^^8^^,^^19^ In our study, only those individuals that received colonoscopy 6 months after piecemeal polypectomy for scar inspection might have endured the same type of bias. Actually, this might be the reason for the lower HR of individuals with large lesions than expected based on these long-term studies. Lacking information on death or loss of follow-up might have caused that a small proportion of individuals was erroneously not censored in our study. However, due to the relatively short median follow-up time and large number of individuals we expect this to not limit the results. Furthermore, the FIT-population might have been biased as FIT selects individuals at higher risk for adenomas but not serrated polyps.^16^^,^^20^ Nevertheless, this selection bias is unlikely to affect our study endpoint, which is the association with metachronous CRC using a negative colonoscopy as comparator.

High-risk serrated polyps are currently being recognized as high-risk lesions in many post-polypectomy guidelines: those issued by the European Society of Gastrointestinal Endoscopy (ESGE), the US Multi-society Task Force on Colorectal Cancer (UMSTFC), and the British Society of Gastroenterology (BSG). High-risk serrated polyps are uniformly defined as any serrated polyp ≥10 mm, SSL with dysplasia, or TSA. Our data confirmed this increased risk of individuals that had any high-risk serrated polyp without co-existing high-risk adenomas as well as individuals with concurrent high-risk adenomas (HR 1.702, 1.078–2.686 and HR 2.003, 1.185–3.387). Looking into the subgroups of high-risk serrated polyps this risk seems largely depending on presence of any TSA, since this was an independent risk factor (HR 2.089). Other studies reported increased CRC risks for all of high-risk subtypes,^7^^,^^9^^,^^12^^,^^19^^,^^21^ although only one study took into account the presence of other co-existing lesions.^8^ Since we also found an increased risk for SSLs with dysplasia in the univariate analysis, this CRC risk might have been partially caused by the incidence of concurrent lesions.

The metachronous risk of CRC after resection of high-risk adenomas has been studied more profoundly than that for high-risk serrated polyps.^7^^,^^22^, ^23^, ^24^ The established evidence for this increased metachronous risk led to a 3-year surveillance advice in current post-polypectomy guidelines for large adenomas and adenomas with high-grade dysplasia.^3^, ^4^, ^5^ In our primary analysis we did not find an increased risk for individuals that had an high-risk adenoma in absence of high-risk serrated polyps. The risk was increased however when an high-risk adenoma was present in combination with an high-risk serrated polyp. Additionally, secondary analyses showed an increased risk for the subgroup of individuals that had adenomas with high grade dysplasia, but not for those individuals with large adenomas (≥10 mm and ≥20 mm) or 5 or more low-risk adenomas. The majority of high-risk adenomas in our study were large adenomas (≥10 mm). Therefore, large adenomas without HGD could have decreased the overall risk of individuals with high-risk adenomas. An explanation for the lack of association between large adenomas and metachronous CRC could be that large pedunculated adenomas are more easily resected *en bloc* and thus more often resected complete as compared to large serrated polyps, that are more challenging to resect completely as demonstrated in a previous study.^25^ In case of large serrated polyps and large adenomas, it might therefore be reasonable that the serrated subtype attributes more to the eventual CRC risk. Furthermore, most large adenomas that are resected piecemeal receive a colonoscopy for scar inspection within 6–12 months (as discussed above), decreasing the risk for a metachronous CRC by removal of residual polyp tissue if present, and by removal of other polyps that have been missed at baseline colonoscopy. Of note, exclusion of patients that were referred for additional treatment might have underestimated the frequency of high-risk adenomas, however, a different prevalence is not likely to affect associations. Future validation of adenoma size as independent risk factor for CRC is warranted to provide more evidence for new post-polypectomy guidelines.

Another remarkable finding of our study was that villous adenomas had adjusted increased risk for metachronous CRC (HR 2.069, 1.341–3.190). Although villous adenomas are not taken into account as high-risk adenomas in the recent ESGE post-polypectomy guideline, our data support stringent surveillance for individuals with villous adenomas.

Our data confirm results from previous studies showing no increased metachronous CRC risk after polypectomy of low-risk serrated polyps. In our model that accounted for co-occurring polyps, the HR for some subgroups (HP < 10 mm, without SSLs, TSAs, or adenomas; low-risk SSL without adenomas; low-risk adenomas with low-risk SSL) could not be evaluated due to the limited number of CRCs, but low overall CRC incidence demonstrated a negligible risk of low-risk serrated polyps. Besides, metachronous CRC incidence was low in individuals with multiplicity of low-risk SSLs (5 or more), although the number of individuals in this group was small (n = 128). Moreover, a considerable number of those individuals should in clinical practice be diagnosed with serrated polyposis syndrome and therefore receive frequent surveillance colonoscopies resulting in a very low risk to develop CRC.^26^^,^^27^ In contrast to the ESGE guideline, the USMSTF advices surveillance colonoscopies in individuals with multiplicity of low-risk SSLs, i.e. 1–2 lesions surveillance after 5–10 years, 3–4 lesions surveillance after 3–5 years, and 5–10 lesions surveillance after 3 years. Our study does not support these surveillance colonoscopies after a high-quality baseline colonoscopy.

Guideline recommendations for surveillance intervals of low-risk adenomas vary from no surveillance (i.e. referral to screening program) in case of <5 polyps (ESGE & BSG) to 7–10 years for 1–2 polyps and 3–5 years for 3–4 polyps (USMSTF). Our results showed no increase in CRC risk for individuals with 1–4 low-risk adenomas without low-risk SSLs in our first model and no increased risk in 1–4 or 3–4 low-risk adenomas evaluated with our second model. In contrast, the subgroup of individuals with ≥5 low-risk adenomas had an increased risk to develop CRC in univariate analysis, but not in multivariate analysis. Therefore, our data provide evidence that a multitude of low-risk adenomas might not be an independent predictor for metachronous CRC risk.

Another interesting finding was the fact that the risk after removal of serrated polyps was especially increased for proximal and not for distal CRCs. The association between serrated polyps and proximal CRC has been well established. Individuals with proximally located serrated polyps and proximal adenomas at baseline colonoscopy showed at univariate analysis to be at increased risk for metachronous proximal CRC. Previous studies demonstrated that interval CRCs after negative FIT, as well as post-colonoscopy CRCs more often originate from a serrated polyp located in the proximal colon.^28^^,^^29^ In fact, proximal CRCs were overrepresented (53.0% proximal vs 44.4% distal) in this cohort with a median follow up duration of 3 years. Given the fact that studies with a longer follow-up tend to represent more distal CRCs, these results suggest that within this short timeframe the role of the serrated neoplasia pathway is more pronounced. This was previously described as a triple threat to post-colonoscopy cancer: SSLs are easily missed and incomplete resected at baseline colonoscopy, and have a relatively short dwell time to CRC once the SSLs contains dysplasia.^30^

In conclusion, we found that high-risk serrated polyps and co-occurring high-risk adenomas, as well as high-risk serrated polyps in absence of high-risk adenomas gave an increased risk to develop metachronous CRC within a median of three years after the baseline colonoscopy for a positive FIT. Adenomas ≥10 mm or ≥20 mm, both previously considered as high-risk feature, did not show an increased risk of CRC in our study setting, while the presence of HGD seems of clinical importance. Our results suggest that individuals with high-risk serrated polyps might comprise the higher CRC risk in the first years after colonoscopy. More focus on improving detection and complete resection of high-risk serrated polyps might reduce this CRC risk.

## Contributors

Study conception & Design: ED, JIJ, DT; Acquisition of data: DT; Data analysis: DT, JIJ, ED; Interpretation of data:DT, JIJ, ED, MS, IN, MVL, IL; Drafting of the manuscript: DT, JIJ; Critical revision of the manuscript for intellectual content: DT, JIJ, ED, MS, IN, MVL, IL; Statistical analysis: DT, JIJ.

## Data sharing statement

Data are available on request from ED.

## Declaration of interests

ED: Received endoscopic equipment on loan of Olympus and FujiFilm and research grant from FujiFilm; received honorarium for consultancy from FujiFilm, Tillots, Olympus, GI Supply, Cancer Prevention Pharmaceuticals, PAION and Ambu; and a speakers' fee from Olympus, Roche, GI Supply, PAION and IPSEN. MS: received research support from Sentinel, Sysmex, Boston Scientific, Norgine and Medtronic. *All other authors have nothing to disclose.*
