# Supplementary material for: Colorectal cancer risk after removal of polyps in fecal immunochemical test based screening
Source: eClinicalMedicine. 2023 Jul 5;61:102066. doi: 10.1016/j.eclinm.2023.102066 (PMC10388570; doi:10.1016/j.eclinm.2023.102066)
Supplement: Supplementary Tables S1–S4 [file mmc1.docx]

**Supplementary**

**Table S1 proximal CRCs**

|  | **Count of individuals** | **Cases of proximal CRC** | **Cases per 1000 person-years of follow-up** | **Univariate HR (95% CI)** | **p-value** | **Multivariate HR (95% CI)*** | **p-value** |
| --- | --- | --- | --- | --- | --- | --- | --- |
| HP <10mm | 30,580 | 47 | 0·49 | 1·641 (1·198-2·248) | 0·002 | 1·382 (0·988-1·932) | 0·059 |
| SSL <10mm, no dysplasia | 10,521 | 14 | 0·46 | 1·545 (0·902-2·647) | 0·113 |  |  |
| SSL low-risk 1-4 | 10,393 | 14 | 0·47 | 1·566 (0·914-2·683) | 0·102 |  |  |
| SSL low-risk ≥5 | 128 | 0 | NA | NA | NA |  |  |
| Low-risk adenoma(s) 1-4 | 93,715 | 96 | 0·32 | 1·006 (0·784-1·292) | 0·960 |  |  |
| Low-risk adenomas 3-4 | 15,439 | 18 | 0·28 | 1·189 (0·737-1·918) | 0·479 |  |  |
| Proximal serrated polyp | 29,161 | 50 | 0·55 | 1·936 (1·424-2·633) | <0·001 | 1·404 (0·984-2·002) | 0·061 |
| Proximal adenoma | 86,178 | 134 | 0·46 | 1·978 (1·556-2·514) | <0·001 | 1·754 (1·367-2·251) | <0·001 |
| Tubular adenoma | 145,028 | 164 | 0·34 | 1·156 (0·904-1·480) | 0·248 |  |  |
| Tubulovillous adenoma | 54,806 | 83 | 0·41 | 1·347 (1·039-1·746) | 0·024 | 1·120 (0·855-1·467) | 0·411 |
| Villous adenoma | 3,427 | 14 | 1·04 | 3·148 (1·837-5·393) | <0·001 | 2·524 (1·462-4·356) | <0·001 |
|  |  |  |  |  |  |  |  |
| **High-risk polyps** |  |  |  |  |  |  |  |
| Serrated polyp ≥10mm | 9,829 | 18 | 0·56 | 1·795 (1·113-2·896) | 0·017 | 1·107 (0·647-1·895) | 0·711 |
| SSL with dysplasia | 2,911 | 8 | 0·82 | 2·631 (1·302-5·317) | 0·007 | 1·632 (0·779-3·420) | 0·194 |
| Traditional serrated adenoma | 2,125 | 8 | 1·09 | 3·360 (1·663-6·791) | <0·001 | 2·503 (1·189-5·270) | 0·016 |
| Adenoma ≥10mm | 69,331 | 84 | 0·34 | 1·022 (0·789-1·324) | 0·867 |  |  |
| Adenoma with HGD | 9,796 | 24 | 0·65 | 2·038 (1·340-3·101) | <0·001 | 1·636 (1·059-2·528) | 0·027 |
| ≥5 low-risk adenomas | 5,815 | 10 | 0·55 | 1·792 (0·953-3·371) | 0·070 |  |  |

HP hyperplastic polyp, SSL sessile serrated lesion, HGD high-grade dysplasia

*adjusted for age at baseline colonoscopy, sex, and other significant subgroups at univariate analysis

**Table S2 distal CRCs**

|  | **Count of individuals** | **Cases of distal CRC** | **Cases per 1000 person-years of follow-up** | **Univariate HR (95% CI)** | **p-value** | **Multivariate HR (95% CI)*** | **p-value** |
| --- | --- | --- | --- | --- | --- | --- | --- |
| HP <10mm | 30,580 | 25 | 0·26 | 0·957 (0·632-1·451) | 0·837 |  |  |
| SSL <10mm, no dysplasia | 10,521 | 5 | 0·17 | 0·638 (0·263-1·548) | 0·320 |  |  |
| SSL low-risk 1-4 | 10,393 | 5 | 0·17 | 0·647 (0·266-1·569) | 0·335 |  |  |
| SSL low-risk ≥5 | 128 | 0 | NA | NA | NA |  |  |
| Low-risk adenoma 1-4 | 93,715 | 81 | 0·27 | 1·005 (0·766-1·320) | 0·969 |  |  |
| Low-risk adenoma 3-4 | 15,439 | 15 | 0·23 | 1·173 (0·695-1·981) | 0·550 |  |  |
| Proximal serrated polyp | 29,161 | 20 | 0·22 | 0·818 (0·517-1·295) | 0·391 |  |  |
| Proximal adenoma | 86,178 | 86 | 0·30 | 1·207 (0·923-1·579) | 0·169 |  |  |
| Tubular adenoma | 145,028 | 139 | 0·29 | 1·163 (0·890-1·520) | 0·269 |  |  |
| Tubulovillous adenoma | 54,806 | 66 | 0·33 | 1·223 (0·918-1·630) | 0·169 |  |  |
| Villous adenoma | 3,427 | 7 | 0·52 | 1·807 (0·851-3·836) | 0·123 |  |  |
|  |  |  |  |  |  |  |  |
| **High-risk polyps** |  |  |  |  |  |  |  |
| Serrated polyp ≥10mm | 9,829 | 9 | 0·28 | 1·032 (0·530-2·010) | 0·927 |  |  |
| SSL with dysplasia | 2,911 | 3 | 0·31 | 1·140 (0·365-3·563) | 0·821 |  |  |
| Traditional serrated adenoma | 2,125 | 3 | 0·40 | 1·458 (0·467-4·555) | 0·516 |  |  |
| Adenoma ≥10mm | 69,331 | 72 | 0·29 | 1·039 (0·785-1·375) | 0·787 |  |  |
| Adenoma with HGD | 9,796 | 29 | 0·79 | 3·020 (2·045-4·461) | <0·001 | 2·841 (1·922-4·201) | <0·001 |
| ≥5 low-risk adenomas | 5,815 | 10 | 0·55 | 2·141 (1·136-4·037) | 0·019 | 1·845 (0·977-3·487) | 0·059 |

HP hyperplastic polyp, SSL sessile serrated lesion, HGD high grade dysplasia

*adjusted for age at baseline colonoscopy, sex, and other significant subgroups at univariate analysis

**Table S3 sensitivity analysis excluding 12 months in primary analysis**

|  | **Count of individuals** | **Count of CRC cases** | **Cases per 1000 person-years of follow-up** | **Unadjusted HR** | **p-value** | **Adjusted HR*** | **p-value** |
| --- | --- | --- | --- | --- | --- | --- | --- |
| No polyps | 70,458 | 105 (0·1%) | 0·48 | ref |  | ref |  |
| HP <10mm,  without SSLs, TSAs, or adenomas | 5,299 | 5 (0·1%) | 0·32 | NA | NA | NA | NA |
| Low-risk SSL without adenomas | 1,865 | 1 (0·1%) | 0·20 | NA | NA | NA | NA |
| Low-risk adenomas without low-risk SSL | 38,901 | 43 (0·1%) | 0·36 | 0·761 (0·533-1·085) | 0·131 | 0·755 (0·529-1·080) | 0·124 |
| Low-risk adenomas with low-risk SSL | 1,708 | 3 (0·2%) | 0·66 | NA | NA | NA | NA |
| High-risk adenoma without high-risk serrated polyp | 70,625 | 149 (0·2%) | 0·59 | 1·137 (0·886-1·460) | 0·314) | 1·146 (0·889-1·476) | 0·667 |
| High-risk serrated polyp without high-risk adenoma | 7,827 | 20 (0·3%) | 0·81 | 1·669 (1·034-2·692) | 0·036 | 1·688 (1·046-2·724) | 0·032 |
| High-risk serrated polyp with high-risk adenoma | 4,203 | 15 (0·4%) | 1·08 | 1·940 (1·129-3·333) | 0·016 | 2·011 (1·168-3·463) | 0·012 |

HP hyperplastic polyp, SSL sessile serrated lesion, TSA traditional serrated adenoma

*adjusted for age at baseline colonoscopy and sex

**Table S4 sensitivity analysis excluding 12 months in secondary analysis**

|  | **Count of individuals** | **Cases of CRC** | **Cases per 1000 person-years of follow-up** | **Univariate HR (95% CI)** | **p-value** | **Multivariate HR (95% CI)*** | **p-value** |
| --- | --- | --- | --- | --- | --- | --- | --- |
| HP <10mm | 30,573 | 62 | 0·64 | 1·239 (0·948-1·620) | 0·117 |  |  |
| SSL <10mm, no dysplasia | 10,520 | 18 | 0·60 | 1·137 (0·718-1·801) | 0·583 |  |  |
| SSL low-risk 1-4 | 10,392 | 18 | 0·60 | 1·215 (0·758-1·948) | 0·418 |  |  |
| SSL low-risk ≥5 | 128 | 0 | NA | NA | NA |  |  |
| Low-risk adenoma 1-4 | 93,689 | 152 | 0·51 | 0·924 (0·760-1·124) | 0·430 |  |  |
| Low-risk adenoma 3-4 | 15,430 | 25 | 0·39 | 0·978 (0·653-1·464) | 0·915 |  |  |
| Proximal serrated polyp | 29,154 | 63 | 0·70 | 1·386 (1·062-1·810) | 0·016 | 1·212 (0·916-1·604) | 0·179 |
| Proximal adenoma | 86,150 | 195 | 0·67 | 1·509 (1·252-1·819) | <0·001 | 1·328 (1·091-1·617) | 0·005 |
| Tubular adenoma | 144,990 | 269 | 0·56 | 1·083 (0·897-1·308) | 0·408 |  |  |
| Tubulovillous adenoma | 54,790 | 136 | 0·68 | 1·268 (1·036-1·551) | 0·021 | 1·096 (0·88-1·352) | 0·394 |
| Villous adenoma | 3,425 | 20 | 1·49 | 2·581 (1·649-4·042) | <0·001 | 2·141 (1·360-3·373) | 0·001 |
|  |  |  |  |  |  |  |  |
| **High-risk polyps** |  |  |  |  |  |  |  |
| Serrated polyp ≥10mm | 9,827 | 25 | 0·78 | 1·466 (0·979-2·194) | 0·063 |  |  |
| SSL with dysplasia | 2,910 | 10 | 1·04 | 1·934 (1·033-3·620) | 0·039 | 1·491 (0·778-2·858) | 0·228 |
| Traditional serrated adenoma | 2,124 | 10 | 1·35 | 2·453 (1·311-4·591) | 0·005 | 2·117 (1·125-3·983) | 0·020 |
| Adenoma ≥10mm | 69,314 | 142 | 0·57 | 1·011 (0·828-1·233) | 0·917 |  |  |
| Adenoma HGD | 9,785 | 42 | 1·15 | 2·084 (1·517-2·864) | <0·001 | 1·768 (1·270-2·459) | <0·001 |
| ≥5 low-risk adenomas | 5,812 | 17 | 0·94 | 1·833 (1·129-2·976) | 0·014 | 1·434 (0·874-2·353) | 0·153 |

HP hyperplastic polyp, SSL sessile serrated lesion, HGD high grade dysplasia

*adjusted for age at baseline colonoscopy, sex, and other significant subgroups at univariate analysis
